# A Low-Dimensional Network Model for an SIS Epidemic: Analysis of the Super Compact Pairwise Model

**DOI:** 10.1007/s11538-021-00907-2

**Published:** 2021-05-21

**Authors:** Carl Corcoran, Alan Hastings

**Affiliations:** 1 Department of Mathematics, University of California, Davis, USA; 2 Department of Environmental Science and Policy, University of California, Davis, USA; 3 Santa Fe Institute, Santa Fe, USA

**Keywords:** SIS epidemic, Pairwise model, Epidemic threshold, Endemic equilibrium

## Abstract

Network-based models of epidemic spread have become increasingly popular in recent decades. Despite a rich foundation of such models, few low-dimensional systems for modeling SIS-type diseases have been proposed that manage to capture the complex dynamics induced by the network structure. We analyze one recently introduced model and derive important epidemiological quantities for the system. We derive the epidemic threshold and analyze the bifurcation that occurs, and we use asymptotic techniques to derive an approximation for the endemic equilibrium when it exists. We consider the sensitivity of this approximation to network parameters, and the implications for disease control measures are found to be in line with the results of existing studies.

## Introduction

In the past few decades, network-based models of epidemic spread have become a central topic (Kiss et al. 2017; Pastor-Satorras et al. 2015) in epidemiology. Their ability to capture mathematically the complex structure of transmission interactions makes them an invaluable theoretical paradigm. Mathematically, a network is modeled as a graph consisting of a set of nodes that are connected by a set of links (called edges). In the context of epidemiology, typically nodes represent individuals, and edges represent interactions that can transmit the infection. Used in conjunction with compartment models, the disease natural history determines the number of possible states an individual node might be in at any point in time. When disease spread is modeled as a continuous time Markov chain, the network size and disease natural history can lead to high dimensional state spaces. For example, in a network with *N* nodes where individual nodes can be in *m* possible states, the size of the state space for the network is $m^N.$ Efforts to describe this process with a system of ordinary differential equations are similarly hampered by size—the Kolmogorov equations governing this system are exact, but prohibitively large. Thus, an important goal in network-based modeling has been to find a (relatively) low-dimensional system that accurately approximates the underlying high-dimensional system.

Many approaches (Pastor-Satorras and Vespignani 2001; Pastor-Satorras et al. 2015; Miller et al. 2012; Barnard et al. 2019; Karrer and Newman 2010) in recent years have sought to introduce models with systems of a manageable size. Pairwise models (Keeling 1999; Eames and Keeling 2002; House and Keeling 2011) have been a popular and fruitful approach to this question. Derived from the Kolmogorov equations and exact in their unclosed form (Taylor et al. 2012), pairwise models consider the evolution of not just the expected number of nodes in a given state, but also pairs and triples of nodes. The dynamical variables are of the form [*A*] (the expected number of nodes in state *A*), [*AB*] (the expected number of pairs in state $A-B$), and [*ABC*] (the expected number of triples in state $A-B-C$). Higher-order groupings of nodes are also considered but rarely written, as dimension-reduction efforts often focus on approximating the expected number of triples in terms of pairs and individual nodes. Pairwise models have been successful with a variety of different network types, with models developed for networks with heterogeneous degree (Eames and Keeling 2002), weighted networks (Rattana et al. 2013), directed networks (Sharkey et al. 2006), and networks with motifs (House et al. 2009; Keeling et al. 2016) to name a few. Moreover, pairwise models have been developed for a variety of disease natural histories, with particular focus on SIR (susceptible-infectious-recovered) and SIS (susceptible-infectious-susceptible) models.

In this paper, we consider an SIS pairwise model for networks with heterogeneous degree. SIS dynamics are used to model diseases where no long-term immunity is conferred upon recovery, leading to their frequent application to sexually transmitted infections such as chlamydia or gonorrhea (Eames and Keeling 2002). Contact networks for diseases of this type frequently involve heterogeneity in the number of contacts for individuals, and thus node degree becomes an essential concept. The degree of a node in a network is the number of edges to which the node is connected, and thus the number of potential infectious contacts. In this way, heterogeneous networks can capture complex disease dynamics. An essential tool when working with such networks is the degree distribution, defined by $p_k$ which is the probability a randomly selected node has degree *k*. The degree distribution has played an important role in dimension reduction approximations for pairwise models.

For the SIR-type diseases, accurate low-dimensional models have been derived from the pairwise family using probability generating functions (Miller et al. 2012), complete with conditions for finding the final size of the epidemic. Despite the successes of the SIR case, the dimension reduction techniques in Miller et al. (2012) do not apply to the SIS case. Instead, the development of low-dimensional models of SIS-type disease spread on networks has relied on moment closure approximations. Under the assumption of a heterogeneous network with no clustering, House and Keeling (2011) introduced an approximation reducing the system size from ${\mathcal {O}}(N^2)$ to ${\mathcal {O}}(N)$, where *N* is the number of nodes in the network. Termed the compact pairwise model (CPW), it has shown good agreement with stochastic simulations despite its considerably smaller size. However, the number of model equations still grows as the maximum degree of the network, making its application challenging for large networks with significant degree heterogeneity. Perhaps the most successful model in reducing the number of equations of the CPW for SIS-type diseases is the super compact pairwise model (SCPW) (Simon and Kiss 2016). The system consists of only four equations, with network structure being encoded to the model through the first three moments of the degree distribution. While Simon and Kiss demonstrated excellent agreement between the CPW and the SCPW, bifurcation analysis of the model and an explicit formula for the endemic steady state remains to be done.

This paper sets out on that analysis of the SCPW model. A common point of investigation among models of SIS-type diseases is the disease-free equilibrium (DFE) that loses stability as a relevant parameter passes a critical value known as the epidemic threshold (Pastor-Satorras and Vespignani 2001, 2002; Boguñá and Pastor-Satorras 2002). The epidemic threshold serves as a dividing point between two qualitatively different types of outbreaks. Below the epidemic threshold, any outbreak will die out; above the epidemic threshold, the system converges asymptotically to a stable equilibrium where the disease remains endemic in the population. Many studies follow the “next generation matrix” approach for the basic reproduction number $R_0$ (van den Driessche and Watmough 2002) to characterize the epidemic threshold. We follow a more conventional bifurcation analysis to derive the epidemic threshold and offer a proof that the system undergoes a transcritical bifurcation, as one might expect. Perhaps more importantly, the SCPW’s small fixed number of equations presents an excellent opportunity to investigate the endemic equilibrium for SIS models on heterogeneous networks, which has been heretofore inhibited by large system size. We present a novel asymptotic approach to approximating the endemic equilibrium, leveraging the low-dimensionality of the model. The results presented further our understanding of the SCPW model specifically and suggest potential new avenues in the challenging problem of analytically determining the nontrivial steady state of pairwise models of SIS-type diseases.

The paper is structured as follows: in Sect. 2, we nondimensionalize the model and reduce the number of equations to 3 to facilitate computations. In Sect. 3, we derive the epidemic threshold and show that the system undergoes a forward transcritical bifurcation. In Sect. 4, we tackle the endemic steady state that emerges through the bifurcation. We use asymptotic methods to approximate the size of the endemic steady state under two regimes—the system near the epidemic threshold and the system far away from the epidemic threshold—and give examples of the efficacy of these approximations on prototypical networks. Finally, we examine the implications of these two approximations. In line with existing studies (Eames and Keeling 2002), we find that control measures for reducing the prevalence at the endemic equilibrium may require different tactics depending on the regime.

## Model

Pairwise models of SIS-type diseases provide a network-based analog of the classical SIS model (Diekmann and Heesterbeek 2000).The essential characteristics of pairwise models of SIS epidemics are dynamical equations for not just the expected number of nodes in each state, but also pairs and triples of nodes. At the node level, [*S*] and [*I*] are the expected number of susceptible and infectious nodes, respectively. At the pair level, [*SI*] is the expected number of connected pairs of susceptible and infectious nodes, while [*SS*] and [*II*] are the expected numbers of connected susceptible-susceptible and infectious-infectious pairs, respectively. The full pairwise model (Eames and Keeling 2002) further requires equations for the expected number of triples ([*SSI*] and [*ISI*]) and higher motifs as well:

$$\begin{aligned} \dot{[S]}&= \gamma [I] - \tau [SI], \\ \dot{[I]}&= \tau [SI] - \gamma [I], \\ \dot{[SI]}&= \gamma ([II] - [SI]) +\tau ([SSI]-[ISI]-[SI]), \\ \dot{[SS]}&= 2\gamma [SI]-2\tau [SSI], \\ \dot{[II]}&= -2\gamma [II]+2\tau ([ISI]+ [SI]). \end{aligned}$$

The CPW closes the system by approximating the expected number of triples as

$$\begin{aligned}{}[ASI] \approx [AS][SI]\frac{S_2-S_1}{S_1^2}, \end{aligned}$$

where $S_1$ and $S_2$ are the first and second moments of the distribution of susceptible nodes; that is

$$\begin{aligned} S_1 = \sum _k k[S_k] =[SS] + [SI],\,\, S_2 = \sum _k k^2[S_k], \end{aligned}$$

where $[S_k]$ is the expected number of susceptible nodes with degree *k*. Unfortunately, $S_2$ cannot be expressed exactly in terms of [*S*], [*I*], [*SI*], [*SS*],  and [*II*] only, so the SCPW model offers an approximation that depends on these variables and moments of the degree distribution.

The SCPW model derived in Simon and Kiss (2016) is given as

1 $$\begin{aligned} \dot{[S]}&= \gamma [I] - \tau [SI], \end{aligned}$$

2 $$\begin{aligned} \dot{[I]}&= \tau [SI] - \gamma [I] \end{aligned}$$

3 $$\begin{aligned} \dot{[SI]}&= \gamma ([II] - [SI]) - \tau [SI]+ \tau [SI] ([SS]-[SI])Q, \end{aligned}$$

4 $$\begin{aligned} \dot{[SS]}&= 2\gamma [SI]-2\tau [SI][SS]Q, \end{aligned}$$

5 $$\begin{aligned} \dot{[II]}&= -2\gamma [II]+2\tau [SI]+2\tau [SI]^2Q, \end{aligned}$$

where

$$\begin{aligned} Q = \frac{1}{n_S [S]}\left( \frac{\langle k^2 \rangle (\langle k^2 \rangle - \langle k \rangle n_S)+\langle k^3 \rangle (n_S-\langle k \rangle )}{n_S(\langle k^2 \rangle - \langle k \rangle ^2)}-1\right) ,\, n_S = \frac{[SI]+[SS]}{[S]}, \end{aligned}$$

$\langle k^n \rangle$ is the *n*th moment of the degree distribution, $\tau$ is the transmission rate, and $\gamma$ is the recovery rate. Here, the quantity *Q* serves as an approximation of $(S_2-S_1)/S_1^2.$ As well, the quantities [*S*], [*I*], [*SI*], [*SS*], [*II*] satisfy conservation equations

6 $$\begin{aligned}{}[S] + [I]&= N, \end{aligned}$$

7 $$\begin{aligned} 2[SI] + [SS]+[II]&= \langle k\rangle N . \end{aligned}$$

With the goal of performing bifurcation and asymptotic analyses in mind, nondimensionalizing the SCPW model is a natural first step. To do so, we will rearrange the Eqs. (3)–(5) so that the network parameters $\langle k \rangle , \langle k^2 \rangle , \langle k^3 \rangle$ are consolidated into more workable constants. First, we rewrite *Q* as

8 $$\begin{aligned} Q = \frac{\alpha [S]}{([SI]+[SS])^2}+\frac{\beta }{[SI]+[SS]}, \end{aligned}$$

where

9 $$\begin{aligned} \alpha = \frac{\langle k^2 \rangle ^2-\langle k \rangle \langle k^3 \rangle }{\langle k^2 \rangle - \langle k \rangle ^2},\,\,\, \beta = \frac{\langle k^3 \rangle -\langle k^2 \rangle \langle k \rangle }{\langle k^2 \rangle - \langle k \rangle ^2}-1. \end{aligned}$$

A natural nondimensionalization of this system is to scale the number of nodes and links in each state to the proportion of nodes and pairs in each state: $v = [S]/N, w = [I]/N, x=[SI]/(\langle k \rangle N),y=[SS]/(\langle k \rangle N),z=[II]/(\langle k \rangle N).$ As well, a natural rescaling of time is $T = t/\gamma ,$ which prompts the defining of the transmission-recovery rate ratio $\delta = \tau /\gamma .$ The introduction of $\delta$ consolidates the two epidemiological parameters $\tau$ and $\gamma$ into a single nondimensional parameter, so any changes to epidemiology of the disease will be captured in $\delta$ alone. With these substitutions, the system (1)–(5) becomes

10 $$\begin{aligned} {\dot{v}}&= w - \langle k \rangle \delta x, \end{aligned}$$

11 $$\begin{aligned} {\dot{w}}&= \langle k \rangle \delta x - w, \end{aligned}$$

12 $$\begin{aligned} {\dot{x}}&= z - \left( \delta + 1\right) x+\frac{\alpha \delta }{\langle k \rangle }\cdot \frac{vx(y-x)}{(x+y)^2}+\beta \delta \cdot \frac{x(y-x)}{x+y}, \end{aligned}$$

13 $$\begin{aligned} {\dot{y}}&= 2x - \frac{2\alpha \delta }{\langle k \rangle }\cdot \frac{vxy}{(x+y)^2}-2\beta \delta \cdot \frac{xy}{x+y}, \end{aligned}$$

14 $$\begin{aligned} {\dot{z}}&= -2z+2\delta x+\frac{2\alpha \delta }{\langle k \rangle }\cdot \frac{vx^2}{(x+y)^2}+2\beta \delta \cdot \frac{x^2}{x+y}, \end{aligned}$$

where the dot notation represents the derivative with respect to the nondimensional time variable $\frac{d}{dT}$. The conservation Eqs. (6) and (7) become

15 $$\begin{aligned} v+w&= 1, \end{aligned}$$

16 $$\begin{aligned} 2x+y+z&= 1, \end{aligned}$$

respectively.

At this point, the conservation equations can be used to reduce the system to a mere 3 equations. However, the elimination of different equations for different analyses will be convenient. For characterizing the bifurcation undergone by the disease-free equilibrium (DFE), it is convenient to work with variables that are 0 at the DFE. For approximating the endemic steady state using asymptotic methods, the most parsimonious equations will make the algebraic manipulation required easier. Thus, we will work with slightly different (but equivalent) characterizations of (10)–(14) in the sections that follow.

## Epidemic Threshold

To derive the epidemic threshold, we consider the stability of the DFE in terms of the epidemiological parameter $\delta .$ We will show that as $\delta$ increases through a critical value $\delta _c,$ the DFE loses stability. Typically as the DFE loses stability, an asymptotically stable endemic equilibrium emerges. The SCPW is no exception, and here we derive the epidemic threshold, with a proof that the system undergoes a transcritical bifurcation (and thus an endemic equilibrium emerges) when $\delta = \delta _c$ included in Appendix A.

First, we use the conservation Eqs. (15) and (16) to eliminate Eqs. (10) and (13). The resulting system is

17 $$\begin{aligned} {\dot{w}}&= \langle k \rangle \delta x - w, \end{aligned}$$

18 $$\begin{aligned} {\dot{x}}&= z - \left( \delta + 1\right) x+\frac{\alpha \delta }{\langle k \rangle }\cdot \frac{(1-w)x(1-3x-z)}{(1-x-z)^2}+\beta \delta \cdot \frac{x(1-3x-z)}{1-x-z}, \end{aligned}$$

19 $$\begin{aligned} {\dot{z}}&= -2z+2\delta x+\frac{2\alpha \delta }{\langle k \rangle }\cdot \frac{(1-w)x^2}{(1-x-z)^2}+2\beta \delta \cdot \frac{x^2}{1-x-z}. \end{aligned}$$

Though ostensibly a messier choice of equation reduction, we note that at the DFE, $[I] = [SI] = [II] =0,$ so $w=x=z=0.$ The notation

20 $$\begin{aligned} \dot{\mathbf {x}} = \begin{bmatrix} {\dot{w}}\\ {\dot{x}} \\ {\dot{z}}\\ \end{bmatrix} = \begin{bmatrix} F_1(w,x,z)\\ F_2(w,x,z) \\ F_3(w,x,z) \\ \end{bmatrix} = {\mathbf {F}}({\mathbf {x}}) \end{aligned}$$

will be convenient moving forward. To determine the stability of the DFE, we compute the Jacobian at ${\mathbf {x}} = \mathbf {0}:$

21 $$\begin{aligned} D{\mathbf {F}} = \begin{bmatrix}-1 &{} \langle k \rangle \delta &{} 0 \\ 0 &{} \left( \dfrac{\alpha }{\langle k \rangle }+\beta \right) \delta - (\delta +1)&{} 1 \\ 0 &{} 2\delta &{}-2\\ \end{bmatrix}. \end{aligned}$$

A straightforward computation shows that

22 $$\begin{aligned} \frac{\alpha }{\langle k \rangle } + \beta = \frac{\langle k^2 \rangle -\langle k \rangle }{\langle k \rangle } = {\bar{k}}. \end{aligned}$$

We can write $D{\mathbf {F}}$ as a block triangular matrix as

$$\begin{aligned} D{\mathbf {F}} = \begin{bmatrix}-1 &{} A \\ 0 &{} B\\ \end{bmatrix}, \end{aligned}$$

where the dimensions *A* and *B*, respectively, are $1\times 2$ and $2\times 2$. The properties of determinants of block matrices tell us that the eigenvalues of $D{\mathbf {F}}$ are $-1$ and the eigenvalues of *B*,  which will determine the stability of the DFE.

We appeal here to the trace-determinant theorem, which tells us the eigenvalues $\xi$ of the $2\times 2$ matrix *B* are given by

$$\begin{aligned} \xi = \frac{\text {Tr}(B)}{2} \pm \frac{\sqrt{(\text {Tr}(B))^2-4\,\text {Det}(B)}}{2}. \end{aligned}$$

First, we observe that these eigenvalues are real, as

23 $$\begin{aligned} \text {Tr}(B)^2-4\,\text {Det}(B) = (\delta ({\bar{k}}-1)+1)^2+8\delta , \end{aligned}$$

which is clearly positive. As a consequence, for the DFE to be stable we must have $\text {Tr}(B) <0$ and $\text {Det}(B) >0.$ The determinant can be written

24 $$\begin{aligned} \text {Det}(B) = 2(1-\delta {\bar{k}}) \end{aligned}$$

and is thus positive if and only if $\delta < 1/{\bar{k}}.$ Moreover, if $\delta < 1/{\bar{k}},$ then

$$\begin{aligned} \text {Tr}(B)< ({\bar{k}}-1)/{\bar{k}}-3 = -2-1/{\bar{k}} <0. \end{aligned}$$

Therefore, we conclude that the DFE is stable for $\delta < 1/{\bar{k}}$ and unstable for $\delta > 1/{\bar{k}}.$ Thus, the epidemic threshold is the critical value of the bifurcation parameter $\delta :$

25 $$\begin{aligned} \delta _c = \frac{\langle k \rangle }{\langle k^2 \rangle - \langle k \rangle }. \end{aligned}$$

Notably, this threshold value is identical to that of the CPW as shown in Kiss et al. (2017). However, it remains to be shown that a bifurcation actually does occur here, and that an asymptotically stable endemic steady state emerges. To prove this, we apply a theorem of Castillo-Chavez and Song (2004) in Appendix A. We note that both the CPW and SCPW models are approximations to the true SIS dynamics on a network, so while (25) is a good approximation of the true epidemic threshold, it may not be appropriate in some cases. For instance, (25) is greater than zero for networks with a power law degree distribution ($p_k \sim k^{-d}$) with $d>3$ in the large network limit ($N\rightarrow \infty$). However, exact results show that the true epidemic threshold is zero in the large network limit (Chatterjee and Durrett 2009).

## The Endemic Equilibrium

With the existence of an endemic steady state established, we turn to the question of finding an approximate analytic expression for it. In general, this is a difficult proposition with epidemic models on networks owing to the frequently high-dimensional nature of the dynamical systems. An exact closed-form expression for the endemic equilibrium of the SCPW model requires solving a system of polynomial equations in multiple variables, which we do not attempt here. However, with asymptotic techniques, a workable approximation can be derived for two cases of $\delta$: near the epidemic threshold ($\delta \approx \delta _c$), and far away from it ($\delta>> \delta _c$). We do not have a good approximation in the intermediate case. Two challenges are apparent. First, how to eliminate equations to facilitate asymptotic expansions of the equilibrium and second, the choice of small nondimensional parameter in each case.

Unlike in Sect. 3, the most parsimonious characterization of (10)–(14) is desirable. So we eliminate (11) and (14) with the conservation equations. To promote the finding of a small nondimensional parameter, we rewrite the resulting system using $\delta = \delta _c\cdot \frac{\delta }{\delta _c}$ and incorporate the constants $\sigma = \langle k \rangle \delta _c, \lambda = \alpha \delta _c/\langle k \rangle , \mu = \beta \delta _c.$ With these alterations, the system becomes

26 $$\begin{aligned} {\dot{v}}&= 1-v-\sigma \frac{\delta }{\delta _c}x, \end{aligned}$$

27 $$\begin{aligned} {\dot{x}}&= 1-y-\left( 3+\delta _c\frac{\delta }{\delta _c}\right) x+\lambda \frac{\delta }{\delta _c}\frac{vx(y-x)}{(x+y)^2}+\mu \frac{\delta }{\delta _c}\frac{x(y-x)}{x+y}, \end{aligned}$$

28 $$\begin{aligned} {\dot{y}}&= 2x-2\lambda \frac{\delta }{\delta _c}\frac{vxy}{(x+y)^2}-2\mu \frac{\delta }{\delta _c}\frac{xy}{x+y}. \end{aligned}$$

At the endemic equilibrium, ${\dot{v}} = {\dot{x}} = {\dot{y}} =0.$ We can solve (26) for *v* and substitute into (27) and (28). With some rearrangement of terms and a little algebra (and adding (28) to (27)), we arrive at the system of polynomial equations that determines the endemic steady state:

29 $$\begin{aligned} 0&= \left( \frac{\delta _c}{\delta }\right) ^2(1-y-2x)(x+y)^2-\frac{\delta _c}{\delta }\left( \delta _c x (x+y)^2+\lambda x^2 + \mu x(x+y)\right) \nonumber \\&\qquad +\lambda \sigma x^3 = P(x,y), \end{aligned}$$

30 $$\begin{aligned} 0&=\left( \frac{\delta _c}{\delta }\right) ^2(x+y)^2 -\frac{\delta _c}{\delta }\left( \lambda y+\mu y (x+y)\right) + \lambda \sigma x y = Q(x,y). \end{aligned}$$

Note that in (30), we have dropped a factor of *x* that corresponds to the DFE. For the endemic steady state, we are interested in knowing the prevalence when the system is at equilibrium: $w^*$. We use the following procedure to approximate the solution.

1. Express $\delta _c/\delta$ in terms of a small parameter.
2. Use the Implicit Function Theorem to linearize $P(x,y)=0$ as
  $$\begin{aligned} y \approx {\tilde{y}} - \dfrac{P_x({\tilde{x}},{\tilde{y}})}{P_y({\tilde{x}},{\tilde{y}})}(x-{\tilde{x}}) \end{aligned}$$
  around a point $({\tilde{x}},{\tilde{y}})$ that is mathematically and/or biologically justified for the given regime.
3. Expand *x*, *y*, and other relevant quantities in terms of the small parameter.
4. Substitute the expansions into $Q(x,y)=0$ and obtain a regular perturbation problem and find an asymptotic solution for the equilibrium value *x*, which approximates $x^*$.
5. Apply the relation $w^* = (\delta _c/\delta )^{-1}\sigma x^*$ to obtain an asymptotic series for the prevalence at the endemic equilibrium.

We describe the results of this procedure for each case in the remainder of this section–the details of the computations are included in Appendix B.

### Case 1: Near the Epidemic Threshold ($\delta \approx \delta _c$)

For $\delta \approx \delta _c,$ we choose $\eta = 1-\delta _c/\delta$ as a small parameter. In terms of this small parameter, (29) and (30) become:

31 $$\begin{aligned} 0&= (1-\eta )^2(1-y-2x)(x+y)^2 \nonumber \\&\qquad -(1-\eta )\left( \delta _cx(x+y)^2+\lambda x^2 +\mu x^2(x+y)\right) +\lambda \sigma x^3, \end{aligned}$$

32 $$\begin{aligned} 0&= (1-\eta )^2(x+y)^2 -(1-\eta )\left( \lambda y +\mu y (x+y)\right) +\lambda \sigma xy. \end{aligned}$$

When $\delta \approx \delta _c,$ an endemic steady state has just emerged, so we can view this equilibrium as a small perturbation to the steady state $x=0,\,y=1.$ Linearizing $P(x,y) = 0$ about this point gives

33 $$\begin{aligned} y \approx 1 - \left( 2+\frac{\delta _c}{1-\eta }\right) x. \end{aligned}$$

Expanding

34 $$\begin{aligned} 2+\frac{\delta _c}{1-\eta } = 2+\delta _c\left( 1+\eta +\eta ^2+{\mathcal {O}}\left( \eta ^3\right) \right) , \end{aligned}$$

35 $$\begin{aligned} x^* = x_0 + x_1\eta +x_2\eta ^2 + {\mathcal {O}}\left( \eta ^3\right) , \end{aligned}$$

we have

36 $$\begin{aligned} y&\approx (1-(2+\delta _c)x_0) -(\delta _cx_0+(2+\delta _c)x_1)\eta \nonumber \\&\qquad - (\delta _cx_0+(2+\delta _c)x_2+\delta _cx_1)\eta ^2 +{\mathcal {O}}\left( \eta ^3\right) . \end{aligned}$$

Substituting into (32) and equating coefficients to 0, we find an $\eta$-order expansion of the approximate equilibrium value $x^*$ as

37 $$\begin{aligned} x^* \approx \frac{1}{\lambda \sigma +\mu \delta _c +\mu -\delta _c}\eta +{\mathcal {O}}\left( \eta ^2\right) . \end{aligned}$$

Using the relation $w^* = \frac{\sigma }{1-\eta }x^* = \sigma x^* +{\mathcal {O}}(\eta ),$ we have

38 $$\begin{aligned} w^* \approx \frac{\sigma }{\lambda \sigma +\mu \delta _c +\mu -\delta _c}\eta +{\mathcal {O}}\left( \eta ^2\right) . \end{aligned}$$

**Fig. 1:**
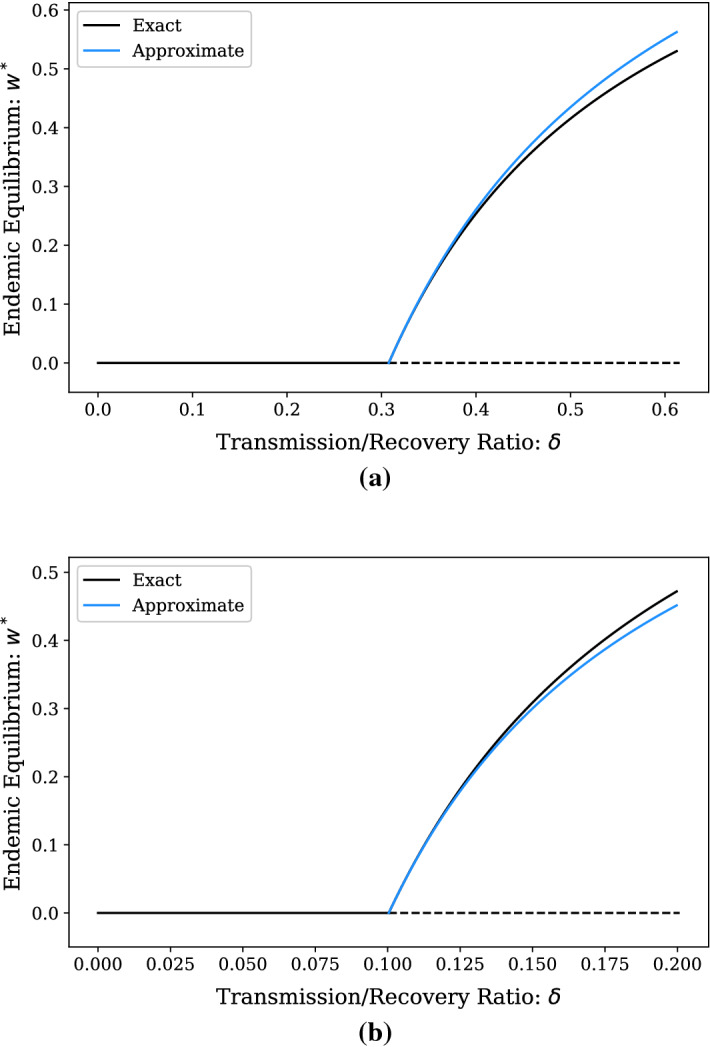
Exact and approximate endemic equilibrium prevalence in the $\delta \approx \delta _c$ regime for **a** a bimodal network with 5000 degree 3 nodes and 5000 degree 5 nodes and **b** a configuration-model network with a Poisson degree distribution with 10,000 nodes and $\langle k \rangle = 10$. Moments of the degree distribution for the bimodal network **a** are $\langle k \rangle = 4, \langle k^2 \rangle = 17, \langle k^3 \rangle = 76$, with $\delta _c = 0.31$, and higher moments of the degree distribution for the Poisson network **b** are $\langle k^2 \rangle \approx 110, \langle k^3 \rangle \approx 1309$, with $\delta _c = 0.1$. Solid lines denote stable equilibria, while dashed lines denote unstable. The equilibrium with $w^*=0$ is the DFE

To demonstrate the efficacy of this approximation, we compare the approximation (38) to the actual endemic equilibrium using bifurcation diagrams (Fig. 1). We consider two example configuration model random networks (Molloy and Reed 1995) with $N=10,000$. In Fig. 1a, a bimodal network is considered with 5000 degree 3 nodes and 5000 degree 5 nodes. In Fig. 1b, a network with a Poisson degree distribution (with average degree $\langle k \rangle =10$) is considered. As is clear in both examples, the agreement between the actual and approximate endemic equilibrium is quite good near the epidemic threshold. Interestingly, the approximate value of $w^*$ is greater than the exact value for the bimodal network and less than the exact value for the Poisson network. We suspect that this is due to network structure and higher order terms in the asymptotic expansion, which we have not computed. An analogous situation is found in the $\delta>> \delta _c$ case.

### Case 2: Far Away from the Epidemic Threshold ($\delta>> \delta _c$)

For $\delta>> \delta _c,$ our small parameter of choice is $\epsilon = \delta _c/\delta$. We can rewrite (29) and (30) in terms of this parameter:

39 $$\begin{aligned} 0&= \epsilon ^2(1-y-2x)(x+y)^2 \nonumber \\&\qquad - \epsilon \left( \delta _cx(x+y)^2+\lambda x^2 +\mu x^2(x+y)\right) +\lambda \sigma x^3, \end{aligned}$$

40 $$\begin{aligned} 0&= \epsilon ^2(x+y)^2 -\epsilon \left( \lambda y +\mu y (x+y)\right) +\lambda \sigma xy. \end{aligned}$$

When $\delta>> \delta _c,$ the transmission rate $\tau$ is large relative to the recovery rate $\gamma .$ Thus, we expect the disease to affect much of the population, and consequently there will be very few remaining [*SS*] links, and therefore $y \approx 0.$

Solving $P(\phi ,0)=0$ for $\phi$ yields

41 $$\begin{aligned} \phi (\epsilon ) = \frac{\epsilon ^2-\lambda \epsilon }{2\epsilon ^2+(\delta _c+\mu )\epsilon -\lambda \sigma }, \end{aligned}$$

and slope of the linearization is then

42 $$\begin{aligned} \psi (\epsilon )=-\frac{P_x(\phi ,0)}{P_y(\phi ,0)} = -\frac{(\epsilon -\lambda )\left( 2\epsilon ^2+(\delta _c+\mu )\epsilon -\lambda \sigma \right) }{\epsilon (\epsilon ^2-(\mu +5\lambda )\epsilon -\lambda (2\delta _c+\mu -2\sigma ))}, \end{aligned}$$

so

43 $$\begin{aligned} y \approx \psi (x-\phi ). \end{aligned}$$

Next, we seek to expand *y* in terms of $\epsilon$ only. The relevant expansions for $\phi ,\psi ,$ and *x* are:

44 $$\begin{aligned} \phi (\epsilon )&= \frac{1}{\sigma }\epsilon + \frac{\delta _c+\mu -\sigma }{\lambda \sigma ^2}\epsilon ^2 + {\mathcal {O}}\left( \epsilon ^3\right) , \end{aligned}$$

45 $$\begin{aligned} \psi (\epsilon )&= \frac{\lambda \sigma }{2\delta _c+\mu -2\sigma }\epsilon ^{-1}-\frac{2\delta _c^2+3\delta _c\mu +\sigma (5\lambda +2\sigma )+\mu ^2}{(2\delta _c+\mu -2\sigma )^2}+{\mathcal {O}}(\epsilon ), \end{aligned}$$

46 $$\begin{aligned} x(\epsilon )&= x_0+x_1\epsilon +x_2\epsilon ^2 + {\mathcal {O}}\left( \epsilon ^2\right) . \end{aligned}$$

To ease the writing of coefficients, we let $\phi _\alpha$ and $\psi _\alpha$ refer to the coefficients on $\epsilon ^\alpha$ for the respective series. From this, it follows that

47 $$\begin{aligned} y&\approx (\psi _{-1}x_0)\epsilon ^{-1} + (\psi _{-1}x_1+\psi _0x_0-\psi _{-1}\phi _1) \nonumber \\&\qquad +(\psi _{-1}x_2+\psi _1x_0+\psi _0x_1-\psi _{-1}\phi _2-\psi _0\phi _1)\epsilon + {\mathcal {O}}\left( \epsilon ^2\right) . \end{aligned}$$

Substituting into (40), and equating the coefficients to 0, we find that we need the coefficients up to order $\epsilon ^4$ in order to find a $\epsilon ^2$ order expansion of the approximate equilibrium value of $x^*.$ The result is

48 $$\begin{aligned} x^* \approx \frac{1}{\sigma }\epsilon + \frac{\delta _c+\mu -\sigma }{\lambda \sigma ^2}\epsilon ^2+{\mathcal {O}}\left( \epsilon ^3\right) . \end{aligned}$$

Finally, as $w^* =\sigma \epsilon ^{-1}x^*,$ we arrive at an $\epsilon -$order approximation for size of the endemic steady state as

49 $$\begin{aligned} w^* \approx 1 + \frac{\delta _c+\mu -\sigma }{\lambda \sigma }\epsilon +{\mathcal {O}}\left( \epsilon ^2\right) . \end{aligned}$$

**Fig. 2:**
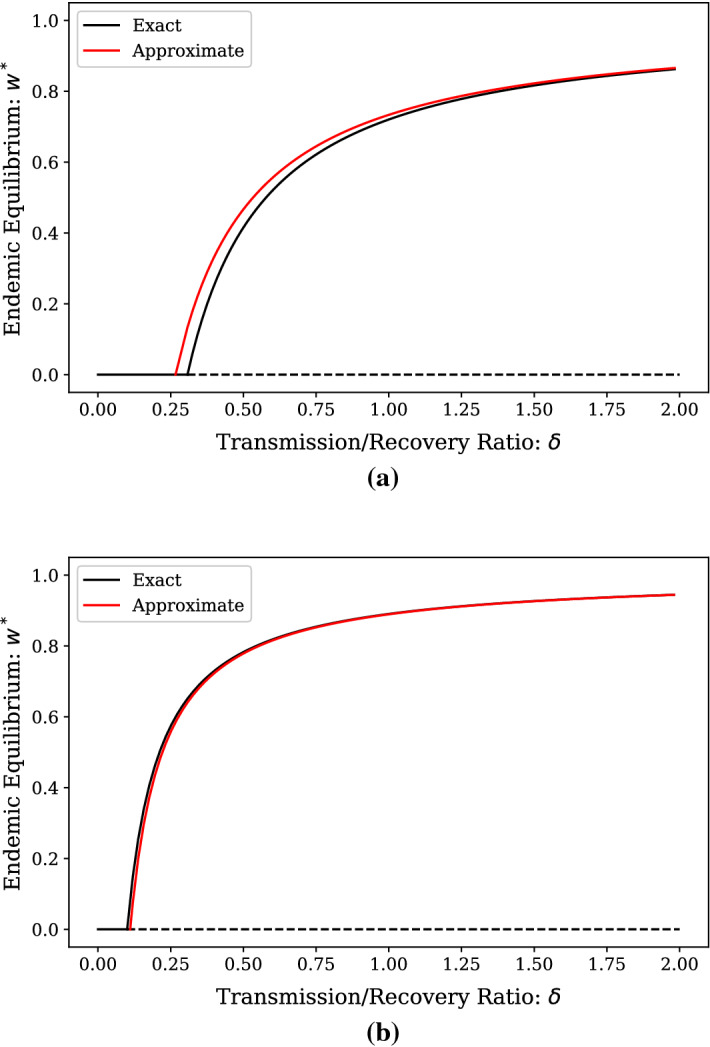
Exact and approximate endemic equilibrium prevalence in the $\delta>> \delta _c$ regime for **a** a bimodal network with 5000 degree 3 nodes and 5000 degree 5 nodes and **b** a configuration-model network with a Poisson degree distribution with 10,000 nodes and $\langle k \rangle = 10$. Moments of the degree distribution for the bimodal network **a** are $\langle k \rangle = 4, \langle k^2 \rangle = 17, \langle k^3 \rangle = 76$, with $\delta _c = 0.31$, and higher moments of the degree distribution for the Poisson network **b** are $\langle k^2 \rangle \approx 110, \langle k^3 \rangle \approx 1309$, with $\delta _c = 0.1$. Solid lines denote stable equilibria, while dashed lines denote unstable. The equilibrium with $w^*=0$ is the DFE

As with the $\delta \approx \delta _c$ case, we compare the approximation (49) to the actual endemic equilibrium in Fig. 2 for the same networks as previously described. Again, the agreement is quite good, even for relatively small values of $\delta .$ In this case, the approximation for the endemic equilibrium also provides an approximation to the epidemic threshold. Whether this approximation is an overestimate or underestimate of the exact threshold depends on network structure. If $\langle k^2 \rangle \ge \langle k \rangle ^2+\langle k \rangle ,$ the approximation is an overestimate. On the other hand, if $\langle k^2 \rangle < \langle k \rangle ^2+\langle k \rangle ,$ the approximation being an overestimate or underestimate depends on the relationship between $\langle k^3 \rangle$ and the other two moments.

### Sensitivity Analysis

With any model of infectious disease, its implications in preventing or mitigating spread should be considered. For network models, some pharmaceutical and non-pharmaceutical interventions can alter the contact network structure in the effort to contain or mitigate outbreaks (Salathé and Jones 2010). For an SIS-type disease, particularly when containment is impossible, one such goal may be to decrease the size of the endemic equilibrium. To that end, we examine the sensitivity of our approximations of $w^*$ to network parameters in the SCPW model. One benefit of explicit asymptotic expressions for the endemic equilibrium is that sensitivity analyses are straightforward to implement.

**Table 1:** Partial derivatives for $\delta \approx \delta _c$

$\left. \displaystyle \frac{\partial w^*}{\partial \langle k \rangle }\right| _{\delta = \delta _c}$ = $\displaystyle -\frac{\langle k^2 \rangle }{\langle k \rangle - 2\langle k^2 \rangle +\langle k^3 \rangle }$	
$\left. \displaystyle \frac{\partial w^*}{\partial \langle k^2 \rangle }\right| _{\delta = \delta _c}$ = $\displaystyle \frac{\langle k \rangle }{\langle k \rangle - 2\langle k^2 \rangle +\langle k^3 \rangle }$	
$$\left. \displaystyle \frac{\partial w^*}{\partial \langle k^3 \rangle }\right| _{\delta =\delta _c}$$ = 0

**Table 2:** Partial derivatives for $\delta>>\delta _c$

$\displaystyle \frac{\partial w^*}{\partial \langle k \rangle }$ = $\displaystyle \frac{\langle k^3 \rangle ^2+3\langle k \rangle ^2\langle k^2 \rangle ^2-2\left( \langle k \rangle ^3\langle k^3 \rangle +\langle k^2 \rangle ^3\right) }{\left( \langle k^2 \rangle ^2 - \langle k^3 \rangle \langle k \rangle \right) ^2}\frac{1}{\delta }$	
$\displaystyle \frac{\partial w^*}{\partial \langle k^2 \rangle }$ = $\displaystyle -\frac{2\left( \langle k \rangle ^2-\langle k^2 \rangle \right) \left( \langle k \rangle \langle k^2 \rangle - \langle k^3 \rangle \right) }{\left( \langle k^2 \rangle ^2 - \langle k^3 \rangle \langle k \rangle \right) ^2}\frac{1}{\delta }$	
$\displaystyle \frac{\partial w^*}{\partial \langle k^3 \rangle }$ = $\displaystyle \frac{\left( \langle k \rangle ^2-\langle k^2 \rangle \right) ^2}{\left( \langle k^2 \rangle ^2 - \langle k^3 \rangle \langle k \rangle \right) ^2}\frac{1}{\delta }$	

For a fixed $\delta ,$ we have a three-dimensional parameter space. To visualize these parameter combinations, we use two-dimensional heat maps taken at slices of the third network parameter. In this case, we have decided to look at several fixed values of $\langle k^3 \rangle$ and draw sensitivity heat maps in the variables $(\langle k \rangle ,\langle k^2 \rangle ).$ Further complicating matters is the fact that moments of a distribution are subject to many inequalities which restrict the domain of the sensitivity heat maps. Two natural restrictions to include are the results of Jensen’s Inequality and the Cauchy–Schwarz Inequality, respectively:

$$\begin{aligned} \langle k^2 \rangle&\ge \langle k \rangle ^2, \\ \langle k^2 \rangle ^2&\le \langle k^3 \rangle \langle k \rangle . \end{aligned}$$

For a fixed value of $\langle k^3 \rangle ,$ these restrictions give a wedge-shaped feasible region of $(\langle k \rangle , \langle k^2 \rangle ).$ We plot the sensitivities for $\langle k^3 \rangle = 20, 100,$ and 400 to display a range of possible parameter combinations.

**Fig. 3:**
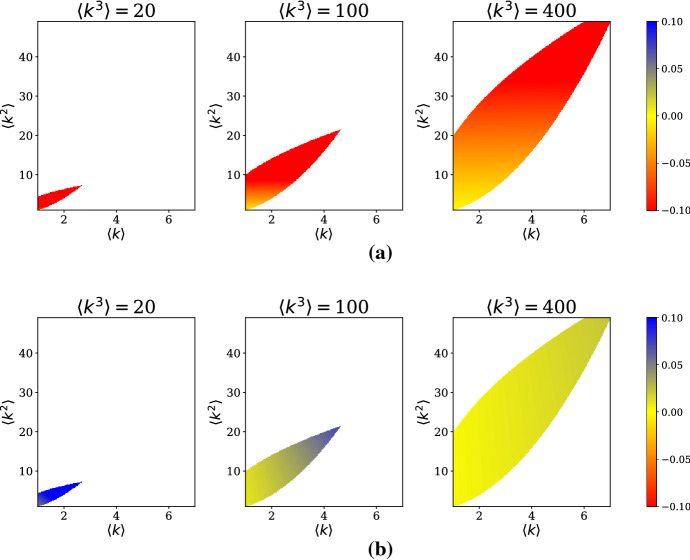
Sensitivities **a** $\frac{\partial w^*}{\partial \langle k \rangle }$ and **b** $\frac{\partial w^*}{\partial \langle k^2 \rangle }$ for the $\delta \approx \delta _c$ approximation. White denotes regions of the $(\langle k \rangle ,\langle k^2 \rangle )$ plane outside of the feasible region. Sensitivities are evaluated at $\delta =\delta _c$

In the $\delta \approx \delta _c$ case, calculating the partial derivatives is straightforward. To compute the sensitivities, we evaluate the partial derivatives at the epidemic threshold: $\delta = \delta _c$. Table 1 shows the expressions for these sensitivities, and Fig. 3 shows corresponding plots. Clearly $\frac{\partial w^*}{\partial \langle k \rangle } \le 0$ and $\frac{\partial w^*}{\partial \langle k^2 \rangle } \ge 0,$ with more extreme values near the upper-right corner of the feasible region.

**Fig. 4:**
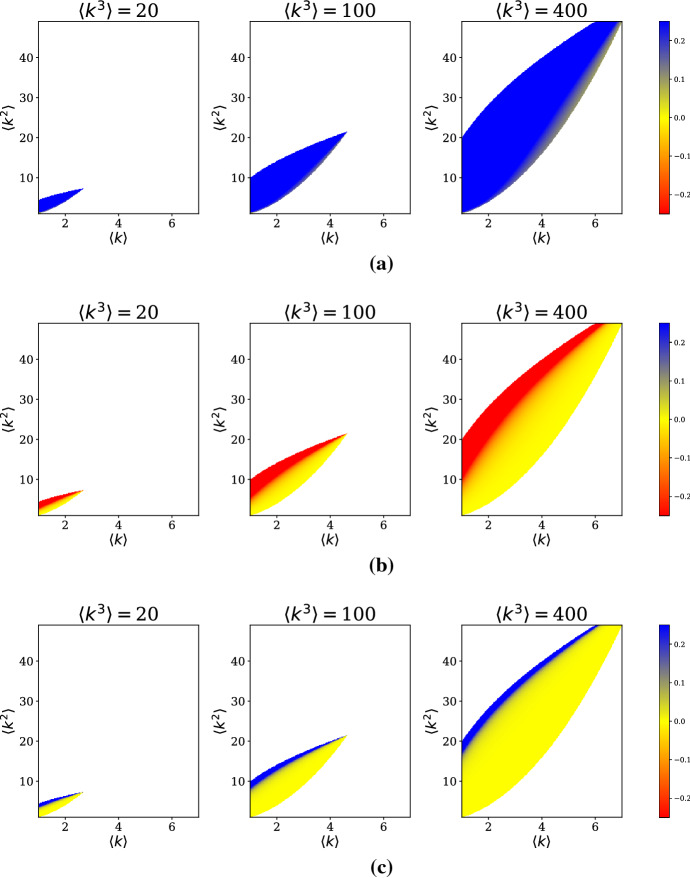
Sensitivities **a** $\frac{\partial w^*}{\partial \langle k \rangle }$, **b** $\frac{\partial w^*}{\partial \langle k^2 \rangle }$, and **c** $\frac{\partial w^*}{\partial \langle k^3 \rangle }$ for the $\delta>> \delta _c$ approximation. White denotes regions of the $(\langle k \rangle ,\langle k^2 \rangle )$ plane outside of the feasible region. Sensitivities are evaluated at $\delta =5\delta _c$

For the $\delta>> \delta _c$ case, the partial derivatives (Table 2) all depend on a factor of $1/\delta ,$ so the choice of $\delta$ for computing sensitivities does not affect the relative magnitudes of the partial derivatives. For convenience, we select $\delta =1.5.$ The sensitivity plots in Fig. 4 show that $\frac{\partial w^*}{\partial \langle k \rangle } \ge 0, \frac{\partial w^*}{\partial \langle k^2 \rangle } \le 0,$ and $\frac{\partial w^*}{\partial \langle k^3 \rangle } \ge 0,$ with the greatest sensitivity near the curve $\langle k^2 \rangle ^2 = \langle k^3 \rangle \langle k \rangle ,$ though the large magnitude appears to be due to the partial derivatives being undefined there.

A significant observation from these sensitivities is that $\frac{\partial w^*}{\partial \langle k \rangle }$ and $\frac{\partial w^*}{\partial \langle k^2 \rangle }$ change signs depending on the regime considered. If the goal of an intervention is to reduce the size of the endemic equilibrium, near the epidemic threshold, this can be accomplished in principle by increasing $\langle k \rangle$ or decreasing $\langle k^2 \rangle ,$ which will in effect increase $\delta _c$ as well. This is intuitive, as an effort to push the system below the epidemic threshold would also decrease the endemic equilibrium for a fixed $\delta$. However, in the $\delta>> \delta _c$ regime, the system is far from the epidemic threshold, and reducing the size of the endemic equilibrium can be accomplished by decreasing $\langle k \rangle$ or increasing $\langle k^2 \rangle .$ This suggests that containment and mitigation strategies that depend on altering the structure of the contact network may require different goals in terms of the moments of the degree distribution.

## Conclusion

In this paper, we have analyzed the super compact pairwise model presented in Simon and Kiss (2016). A non-dimensional version of the model was considered, and a bifurcation analysis was performed, demonstrating that the SCPW and CPW models share an epidemic threshold. Moreover, we derived approximate formulas for the endemic equilibrium in two regimes: when the transmission/recovery ratio is near the epidemic threshold, and far away from it. While the asymptotic techniques used here are ad hoc, similar techniques may prove fruitful in other low-dimensional models of infectious disease spread on networks. However, an exact expression for the endemic equilibrium remains elusive.

Before explaining the advantages of our approach, we acknowledge two limitations of our approximation. First, approximations of the endemic equilibrium for diseases between the two regimes are lacking. Second, while the examples of simulated networks show good agreement between the exact and approximate prevalence, we have not quantified the approximation error generally. As such, there may be types of networks for which our approximation of the endemic equilibrium is less accurate or inappropriate.

Our approximation of the endemic equilibrium is very useful in providing a more detailed look into the interactions of the moments of the degree distribution as they relate to the size of an outbreak. This has implications for disease control measures, particularly those that work by altering the contact network structure. Our results suggest that for SIS-type diseases, strategies to contain (near the epidemic threshold) or mitigate (far away from the epidemic threshold) an outbreak may require different goals. In the mitigation scenario where the prevalence is high, measures might be employed that decrease the first moment $\langle k \rangle$ of the degree distribution. In effect, this may mean initiatives aimed at reducing the number of contacts of individuals alone. On the other hand, in the containment scenario where the prevalence is low, decreasing the second moment $\langle k^2 \rangle$ may be efficient. When couched in degree distribution terms this goal is hard to conceptualize, but using probability generating functions (Newman et al. 2001) one can show that $\langle k^2 \rangle$ is the average number of first and second neighbors of nodes in the network. Thus, measures that reduce both the contacts of individuals and their partners are effective in this scenario. This suggests the importance of contact tracing. We note that the sensitivities also suggest that increasing $\langle k^2 \rangle$ in the high prevalence case and increasing $\langle k \rangle$ in the low prevalence case may lead to a reduction in the size of the endemic equilibrium, though it is not clear why from a biological perspective.

Our results complement the findings of Eames and Keeling (2002), who observed that the effectiveness of two common control measures, screening and contact tracing, depend on the prevalence at the endemic equilibrium. Screening, which targets and treats individuals, is efficient when the prevalence is high. Contact tracing, which targets and treats individuals and their partners, if efficient when the prevalence is low. Unlike this paper, Eames and Keeling implement these measures through epidemiological parameters (rather than through changing network structure). In this way, our results can be viewed as a network-structure analog for their conclusions and confirm that control measures appropriate in a network setting can be found. Further work in this area may include investigating this phenomenon with alternative models of SIS diseases on networks.

## Appendix A Bifurcation and Endemic Steady State

We begin with Theorem 4.1 from Castillo-Chavez and Song (2004), referring to the specific conditions that will be relevant for this analysis. Consider a system of ODEs with a parameter $\phi :$

A.1 $$\begin{aligned} \frac{dx}{dt} = {\mathbf {F}}(x,\phi ),\qquad {\mathbf {F}}:{\mathbb {R}}^n\times {\mathbb {R}}\rightarrow {\mathbb {R}}^n \text { and } {\mathbf {F}}\in C^2\left( {\mathbb {R}}^n\times {\mathbb {R}}\right) . \end{aligned}$$

Assume that 0 is an equilibrium for all values of $\phi .$ Assume further that $D_xf(0,0) = \left( \frac{\partial F_i}{\partial x_j}(0,0)\right)$ is the linearization matrix of (A.1) around the equilibrium 0 and with $\phi =0$, and zero is a simple eigenvalue of this matrix with all other eigenvalues having negative real parts. Assume as well that this matrix has a nonnegative right eigenvector ${\mathbf {w}}$ and left eigenvector ${\mathbf {v}}$ corresponding to the zero eigenvalue. Let $F_k$ be the *k*th component of *f* and

A.2 $$\begin{aligned} a&= \sum _{k,i,j=1}^n v_kw_iw_j\frac{\partial ^2 F_k}{\partial x_i\partial x_j}(0,0), \end{aligned}$$

A.3 $$\begin{aligned} b&= \sum _{k,i=1}^n v_k w_i \frac{\partial ^2 F_k}{\partial x_i\partial \phi }(0,0). \end{aligned}$$

If $a<0$ and $b>0,$ then when $\phi$ changes from negative to positive, 0 changes its stability from stable to unstable. Correspondingly, a negative unstable equilibrium becomes positive and locally asymptotically stable.

We apply this theorem to (17)–(19), where the equilibrium occurs at $w=x=z=0.$ Moreover, we define a bifurcation parameter $\phi = \delta -\delta _c,$ so $\phi = 0$ corresponds to $\delta =\delta _c,$ and $\frac{\partial }{\partial \phi } = \frac{\partial }{\partial \delta }.$ For consistency with previously established notation, we will treat $\delta$ as our parameter, with $\phi$ increasing through 0 as $\delta$ increases through $\delta _c$. The Jacobian given in (21) when $w=0,x=0,z=0,$ and $\delta = \delta _c$ is

A.4 $$\begin{aligned} J = \begin{bmatrix}-1 &{} \langle k \rangle \delta _c &{} 0\\ 0 &{} -\delta _c &{} 1\\ 0 &{}2\delta _c&{}-2\\ \end{bmatrix}. \end{aligned}$$

and the characteristic polynomial is given by

A.5 $$\begin{aligned} 0 = \xi (\xi +1)(\xi - (-2-\delta _c)). \end{aligned}$$

The left and right eigenvectors (${\mathbf {v}}$ and ${\mathbf {w}}$, respectively) corresponding to the eigenvalue $\xi =0$ are

A.6 $$\begin{aligned} {\mathbf {v}} = \begin{bmatrix} 0&2&1\end{bmatrix}, {\mathbf {w}} = \begin{bmatrix} \langle k \rangle&\delta _c^{-1}&1\end{bmatrix}^T. \end{aligned}$$

To compute *a* and *b*, it is convenient to express (A.2) and (A.3) in matrix-vector form:

A.7 $$\begin{aligned} a&= {\mathbf {w}}^T (2H_2 + H_3){\mathbf {w}}, \end{aligned}$$

A.8 $$\begin{aligned} b&= {\mathbf {v}}\frac{\partial J}{\partial \delta }(0,\delta _c) {\mathbf {w}}, \end{aligned}$$

where $H_2$ and $H_3$ are the Hessians of $F_2$ and $F_3$, respectively, at $\mathbf {0}$. These Hessians are

A.9 $$\begin{aligned} H_2 = \begin{bmatrix} 0 &{} -\dfrac{\alpha \delta _c}{\langle k \rangle }&{}0\\ -\dfrac{\alpha \delta _c}{\langle k \rangle } &{} -2-2\beta \delta _c &{} \dfrac{\alpha \delta _c}{\langle k \rangle }\\ 0 &{} \dfrac{\alpha \delta _c}{\langle k \rangle }&{}0\\ \end{bmatrix}, H_3 = \begin{bmatrix} 0&{}0&{}0\\ 0&{}4&{}0\\ 0&{}0&{}0 \end{bmatrix}. \end{aligned}$$

Thus,

A.10 $$\begin{aligned} a&= \begin{bmatrix}\langle k \rangle&\delta _c^{-1}&1 \end{bmatrix}\begin{bmatrix} 0 &{} -\dfrac{2\alpha \delta _c}{\langle k \rangle }&{}0\\ -\dfrac{2\alpha \delta _c}{\langle k \rangle } &{} -4\beta \delta _c &{} \dfrac{2\alpha \delta _c}{\langle k \rangle }\\ 0 &{} \dfrac{2\alpha \delta _c}{\langle k \rangle }&{}0\\ \end{bmatrix}\begin{bmatrix}\langle k \rangle \\ \delta _c^{-1} \\ 1 \end{bmatrix}\nonumber \\&=\begin{bmatrix}\langle k \rangle&\delta _c^{-1}&1 \end{bmatrix}\begin{bmatrix} -\dfrac{2\alpha }{\langle k \rangle }\\ -2\alpha \delta _c-4\beta + \dfrac{2\alpha \delta _c}{\langle k \rangle }\\ \dfrac{2\alpha }{\langle k \rangle }\\ \end{bmatrix}\nonumber \\&= -2\alpha -2\alpha -4\beta /\delta _c+2\dfrac{\alpha }{\langle k \rangle }+2\frac{\alpha }{\langle k \rangle }\nonumber \\&= -4\left( \alpha \left( \frac{1}{\langle k \rangle } +1\right) +\beta \left( \frac{\langle k^2 \rangle }{\langle k \rangle }+1\right) \right) \nonumber \\&=-4\left( \frac{\langle k^3 \rangle }{\langle k \rangle }-1\right) . \end{aligned}$$

As $\langle k^3 \rangle > \langle k \rangle ,$ it follows that $a<0.$

The computation for *b* is simpler. We note that

A.11 $$\begin{aligned} \frac{\partial J}{\partial \delta }(0,\delta _c) = \begin{bmatrix} 0 &{} \langle k \rangle &{} 0 \\ 0 &{} \delta _c^{-1}-1 &{} 0 \\ 0 &{} 2&{} 0\\ \end{bmatrix}. \end{aligned}$$

Thus,

A.12 $$\begin{aligned} b&= \begin{bmatrix} 0&2&1 \end{bmatrix}\begin{bmatrix} 0 &{} \langle k \rangle &{} 0 \\ 0 &{} \delta _c^{-1}-1 &{} 0 \\ 0 &{} 2&{} 0\\ \end{bmatrix}\begin{bmatrix}\langle k \rangle \\ \delta _c^{-1} \\ 1 \end{bmatrix}\nonumber \\&=\begin{bmatrix} 0&2&1 \end{bmatrix}\begin{bmatrix} 0 &{} \langle k \rangle \delta _c^{-1}&{} 0 \\ 0 &{} \delta _c^{-1}(\delta _c^{-1}-1) &{} 0 \\ 0 &{} 2\delta _c^{-1}&{} 0\\ \end{bmatrix} \nonumber \\&= 2\delta _c^{-1}(\delta _c^{-1}-1)+2\delta _c^{-1} = 2\delta _c^{-2} >0. \end{aligned}$$

Finally, as $a<0$ and $b>0,$ we conclude that as $\delta$ increases through $\delta _c,$ a positive, asymptotically stable equilibrium emerges, which is the endemic equilibrium.

## Appendix B Asymptotic Approximations of the Endemic Equilibrium

The full derivations of the approximations (38) and (49) are presented in this appendix.

### Near the Epidemic Threshold ($\delta \approx \delta _c$)

We begin with (31) and (32) and seek the linear approximation of $P(x,y) = 0$ at (0, 1). We compute

B.1 $$\begin{aligned} \frac{\partial P}{\partial x}&= 2(1-\eta )^2\left( (1-y-2x)(x+y)-(x+y)^2\right) \nonumber \\&\qquad -(1-\eta )\left( \delta _c (x+y)(3x+y)+2\lambda x +\mu x (3x+2y)\right) + 3\lambda \sigma x^2, \end{aligned}$$

B.2 $$\begin{aligned} \frac{\partial P}{\partial y}&= (1-\eta )\left( -2\delta _c x(x+y)-\mu x^2+(1-\eta )(x+y)(2-5x-3y)\right) . \end{aligned}$$

The slope of the linear approximation is then

B.3 $$\begin{aligned} \left. -\frac{\partial P/\partial x}{\partial P/\partial y}\right| _{(0,1)} = -\frac{-2(1-\eta )^2-\delta _c(1-\eta )}{-(1-\eta )^2} = -2-\frac{\delta _c}{1-\eta }, \end{aligned}$$

and thus we approximate

B.4 $$\begin{aligned} y \approx 1+\left( -2-\frac{\delta _c}{1-\eta }\right) x. \end{aligned}$$

We now expand *x* as $x = x_0 + x_1\eta +\cdots$ and $\frac{\delta _c}{1-\eta } = \delta _c(1+\eta +\eta ^2+\cdots )$ as a geometric series. Incorporating these with (B.4), we get the approximate expansion of *y* as

B.5 $$\begin{aligned} y&\approx 1 - \left( 2+\delta _c(1+\eta +\eta ^2+\cdots )\right) (x_0 + x_1\eta + x_2\eta ^2\ldots )\nonumber \\&= 1-(2+\delta _c)x_0 - (\delta _c x_0+(2+\delta _c)x_1)\eta \nonumber \\&\qquad -(\delta _c x_0 + \delta _c x_1 + (2+\delta _c)x_2)\eta ^2+\cdots \end{aligned}$$

For easier bookkeeping, define $y_\alpha$ to be the coefficient of $\eta ^\alpha$ in (B.5). As well, the following expansions will prove useful:

B.6 $$\begin{aligned} x^2&= x_0^2 + 2x_0x_1\eta +(x_1^2+2x_0x_2)\eta ^2+\cdots , \end{aligned}$$

B.7 $$\begin{aligned} y^2&= y_0^2 + 2y_0y_1\eta +(y_1^2+2y_0y_2)\eta ^2+\cdots , \end{aligned}$$

B.8 $$\begin{aligned} xy&= x_0y_0 + (x_0y_1+x_1y_0)\eta +(x_0y_2+x_1y_1+x_2y_0)\eta ^2+\cdots \end{aligned}$$

Now, we apply (B.5)–(B.8) to (32) yielding

B.9 $$\begin{aligned} 0&= (1-2\eta +\eta ^2)\left( x_0^2 +2x_0y_0+y_0^2+2(x_0x_1+x_0y_1+x_1y_0 + y_0y_1)\eta + \cdots \right) \nonumber \\&\quad -(1-\eta )\left( \lambda y_0+\mu y_0(x_0+y_0)+(\lambda y_1 +\mu (x_0y_1+x_1y_0+2y_0y_1))\eta +\cdots \right) \nonumber \\&\quad +\lambda \sigma \left( x_0y_0 + (x_0y_1+x_1y_0)\eta +\cdots \right) . \end{aligned}$$

Equating the ${\mathcal {O}}(1)$ terms to zero, we have

B.10 $$\begin{aligned} 0&= x_0^2+2x_0y_0 +y_0^2-\lambda y_0 -\mu y_0(x_0+y_0)+\lambda \sigma x_0y_0 \nonumber \\&= (1-(1+\delta _c)x_0)^2-\lambda (1-(2+\delta _c)x_0)\nonumber \\&\quad -\mu (1-(2+\delta _c)x_0)(1-(1-\delta _c)x_0) \nonumber \\&\quad +\lambda \sigma x_0(1-(2+\delta _c)x_0) \nonumber \\&=1-2(1+\delta _c)x_0+x_0^2-\lambda +\lambda (2+\delta _c)x_0-\mu (1-(3+2\delta _c)x_0) \nonumber \\&\quad -\mu (1+\delta _c)(2+\delta _c)x_0^2+\lambda \sigma x_0-\lambda \sigma (2+\delta _c)x_0^2 \nonumber \\&=\left( 1-\lambda -\mu \right) +\left( \lambda \sigma + \lambda (2+\delta _c)+\mu (3+2\delta _c)-2(1+\delta _c)\right) x_0 \nonumber \\&\quad +\left( 1-\mu (1+\delta _c)(2+\delta _c)-\lambda \sigma (2+\delta _c)\right) x_0^2 \nonumber \\&= x_0\left[ \lambda \sigma + \lambda (2+\delta _c)+\mu (3+2\delta _c)-2(1+\delta _c)\right. \nonumber \\&\quad + \left. \left( 1-\mu (1+\delta _c)(2+\delta _c)-\lambda \sigma (2+\delta _c)\right) x_0\right] . \end{aligned}$$

where we avail ourselves of (22) for the last equality. For the solution were interested, we have $x_0 = 0$ and $y_0 = 1.$

We rewrite (B.9) as

B.11 $$\begin{aligned} 0&= \left( 1-2\eta +\eta ^2\right) \left( 1+(x_1 + 2y_1)\eta + \cdots \right) \nonumber \\&\quad -(1-\eta )\left( \lambda +\mu +(\lambda y_1 +\mu (x_1+2y_1))\eta +\cdots \right) \nonumber \\&\quad +\lambda \sigma \left( x_1\eta +\cdots \right) . \end{aligned}$$

Equating the coefficients of the ${\mathcal {O}}(\eta )$ terms to zero gives

B.12 $$\begin{aligned} 0&= -2+2x_1+2y_1+(\lambda +\mu )-(\lambda y_1+\mu (x_1+2y_1))+\lambda \sigma x_1 \nonumber \\&= -2+2x_1-2(2+\delta _c)x_1+1+\lambda (2+\delta _c)x_1 \nonumber \\&\quad -\mu (x_1-2(2+\delta _c)x_1)+\lambda \sigma x_1 \nonumber \\&= -1 +x_1\left( 2-2(2+\delta _c)+\lambda (2+\delta _c)-\mu (1-2(2+\delta _c))+\lambda \sigma \right) \nonumber \\&= -1 + x_1\left( \lambda \sigma +\mu \delta _c+\mu -\delta _c\right) . \end{aligned}$$

Thus,

B.13 $$\begin{aligned} x_1 = \frac{1}{\lambda \sigma +\mu \delta _c+\mu -\delta _c}. \end{aligned}$$

Now that we have a first-order approximation of *x*,  we obtain an first-order approximation of the endemic equilibrium:

B.14 $$\begin{aligned} w^*&= \frac{\sigma }{1-\eta }x^* \nonumber \\&= \sigma \left( 1+\eta +\eta ^2+\cdots \right) (x_0+x_1\eta +\cdots ) \nonumber \\&= \sigma x_1 \eta + {\mathcal {O}}\left( \eta ^2\right) . \end{aligned}$$

and thus

B.15 $$\begin{aligned} w^* \approx \frac{\sigma }{\lambda \sigma +\mu \delta _c+\mu -\delta _c}\eta + {\mathcal {O}}\left( \eta ^2\right) . \end{aligned}$$

### Far Away from the Epidemic Threshold ($\delta>> \delta _c$)

We begin with (39) and (40) and seek the linear approximation of $P(x,y) = 0$ at $(\phi ,0)$ where $\phi$ is given by (41). We compute

B.16 $$\begin{aligned} \frac{\partial P}{\partial x}&= -2\epsilon ^2(x+y)(3x+2y-1) \nonumber \\&\quad -\epsilon \left( \delta _c\left( 3x^2+4xy+y^2\right) +2\lambda x +\mu x(3x+2y)\right) +2\lambda \sigma x^2, \end{aligned}$$

B.17 $$\begin{aligned} \frac{\partial P}{\partial y}&= \epsilon \left( -2\delta _c x(x+y)-\mu x^2-\epsilon (x+y)(5x+3y-2)\right) . \end{aligned}$$

The slope of the linear approximation is then

B.18 $$\begin{aligned} \left. -\frac{\partial P/\partial x}{\partial P/\partial y}\right| _{(\phi ,0)}&= \frac{-2\epsilon ^2\phi (3\phi -1)-3\epsilon \delta _c\phi ^2+3\lambda \phi + 3\mu \phi ^2+2\lambda \sigma \phi ^2}{\epsilon \left( -2\delta _c\phi ^2-\mu \phi ^2-\epsilon \phi (5\phi -2)\right) }\nonumber \\&= \frac{-(\epsilon ^2-\epsilon \lambda )\phi }{\phi (-\epsilon \phi (2\delta _c+\mu )-\epsilon ^2(5\phi -2))}\nonumber \\&= \frac{-(\epsilon -\lambda )(2\epsilon ^2+\epsilon (\delta _c+\mu )-\lambda \sigma )}{\epsilon \left( \epsilon ^2-\epsilon (\mu +5\lambda )+2\lambda \sigma -\lambda (2\delta _c+\mu )\right) }=\psi (\epsilon ). \end{aligned}$$

Thus, the linear approximation at $(\phi ,0)$ is

B.19 $$\begin{aligned} y \approx \psi (x-\phi ). \end{aligned}$$

We now expand $\psi$ and $\phi$ in powers of $\epsilon :$

B.20 $$\begin{aligned} \psi (\epsilon )&= \psi _{-1}\epsilon ^{-1}+\psi _0 + {\mathcal {O}}(\epsilon )\nonumber \\&=\frac{\lambda \sigma }{2\delta _c+\mu -2\sigma }\epsilon ^{-1}-\frac{2\delta _c^2+3\delta _c\mu +\sigma (5\lambda +2\sigma )+\mu ^2}{(2\delta _c+\mu -2\sigma )^2}+{\mathcal {O}}(\epsilon ), \end{aligned}$$

B.21 $$\begin{aligned} \phi (\epsilon )&= \phi _1\epsilon + \phi _2\epsilon + {\mathcal {O}}\left( \epsilon ^3\right) \nonumber \\&= \frac{1}{\sigma }\epsilon +\frac{\delta _c+\mu -\sigma }{\lambda \sigma ^2}\epsilon ^2+{\mathcal {O}}\left( \epsilon ^3\right) . \end{aligned}$$

Now, we expand *x* as well and reorganize to express *y* as a power series in $\epsilon :$

B.22 $$\begin{aligned} y&\approx \left( \psi _{-1}\epsilon ^{-1}+\psi _0 +\cdots \right) \left( \left( x_0+x_1\epsilon _1+x_2\epsilon ^2+\cdots \right) -\left( \phi _1\epsilon +\phi _2\epsilon ^2+\cdots \right) \right) \nonumber \\&= \psi _{-1}x_0\epsilon ^{-1}+(\psi _{-1}x_1+\psi _0x_0-\psi _{-1}\phi _1)\nonumber \\&\quad +\left( \psi _{-1}x_2+\psi _0x_1+\psi _1 x_0-(\psi _{-1}\phi _2+\psi _0 \phi _1)\right) \epsilon + {\mathcal {O}}\left( \epsilon ^2\right) . \end{aligned}$$

For easier bookkeeping, we define $y_\alpha$ to be the coefficient of $\epsilon ^\alpha$ in (B.22). Again, the following expansions will prove useful:

B.23 $$\begin{aligned} x^2&= x_0^2 + 2x_0x_1\epsilon +\left( x_1^2+2x_0x_2\right) \epsilon ^2+\cdots , \end{aligned}$$

B.24 $$\begin{aligned} y^2&= y_{-1}\epsilon ^{-2} + 2y_{-1}y_0\epsilon ^{-1} +\left( y_0^2+2y_{-1}y_1\right) +\cdots , \end{aligned}$$

B.25 $$\begin{aligned} xy&= x_0y_{-1}\epsilon ^{-1} + (x_0y_0+x_1y_{-1}) +(x_0y_1+x_1y_0+x_2y_{-1})\epsilon +\cdots . \end{aligned}$$

We now apply (B.22)–(B.25) to (40) and multiply by $\epsilon ,$ yielding

B.26 $$\begin{aligned} 0&= \epsilon ^3\left( y_{-1}^2\epsilon ^{-2}+(2y_{-1}y_0+2x_0y_{-1})\epsilon ^{-1} \right. \nonumber \\&\quad +\left( x_0^2 +x_0y_0+x_1y_{-1}+y_0^2+2y_{-1}y_1\right) \nonumber \\&\quad \left. +2\left( x_0x_1+x_0y_1+x_1y_0+x_2y_{-1}+y_{-1}y_2+y_0y_1\right) \epsilon +\cdots \right) \nonumber \\&\quad - \epsilon ^2\left( \mu y_{-1}^2\epsilon ^{-2}+ (\lambda y_{-1}+\mu (x_0y_{-1} +2y_{-1}y_0))\epsilon ^{-1}\right. \nonumber \\&\quad +\lambda y_0 +\mu \left( x_0y_0+x_1y_{-1}+y_0^2+2y_{-1}y_1\right) \nonumber \\&\quad + (\lambda y_1+\mu \left( x_0y_1+x_1y_0+x_2y_{-1}+2y_{-1}y_2+2y_0y_1\right) )\epsilon \nonumber \\&\quad \left. + \left( \lambda y_2+\mu \left( x_0y_2+x_1y_1+x_2y_0+x_3y_{-1}+y_1^2+2y_{-1}y_3+2y_0y_2\right) \right) \epsilon ^2\right. \nonumber \\&\quad \left. + \cdots \right) \nonumber \\&\quad + \epsilon \lambda \sigma \left( x_0y_{-1}\epsilon ^{-1} + (x_0y_0+x_1y_{-1}) +(x_0y_1+x_1y_0+x_2y_{-1})\epsilon \right. \nonumber \\&\quad + (x_0y_2+x_1y_1+x_2y_0+x_3y_{-1})\epsilon ^2 \nonumber \\&\quad +\left. (x_0y_3+x_1y_2+x_2y_1+x_3y_0+x_4y_{-1})\epsilon ^3 \ldots \right) . \end{aligned}$$

Equating the ${\mathcal {O}}(1)$ terms to zero, we have

B.27 $$\begin{aligned} 0 = \lambda \sigma x_0y_{-1}-\mu y_{-1}^2 = x_0^2(\lambda \sigma \psi _{-1}-\mu \psi _{-1}^2), \end{aligned}$$

and thus $x_0 = y_{-1}=0.$ Equating the ${\mathcal {O}}(\epsilon )$ terms to zero, we have

B.28 $$\begin{aligned} 0 = y_{-1}^2+\lambda y_{-1}+\mu (x_0y_{-1}+2y_{-1}y_0)+\lambda \sigma (x_0y_0+x_1y_{-1}), \end{aligned}$$

which is seen to be trivially satisfied as a result of (B.27). Therefore, we look to the ${\mathcal {O}}(\epsilon ^2)$ terms to determine $x_1.$ Equating those coefficients to zero leads to

B.29 $$\begin{aligned} 0&= 2y_{-1}y_0+2x_0y_{-1}-\lambda y_0 -\mu (x_0y_0+x_1y_{-1}+y_0^2+2y_{-1}y_1)\nonumber \\&\qquad +\lambda \sigma (x_0y_1+x_1y_0+x_2y_{-1}) \nonumber \\&= -\lambda y_0 - \mu y_0^2+\lambda \sigma x_1y_0\nonumber \\&= -\psi _{-1}(x_1-\phi _1)(\lambda -\mu \psi _{-1}\phi _1+(\mu \psi _{-1}-\lambda \sigma )x_1). \end{aligned}$$

Of the two solutions to this equation, we are interested in $x_1 = \phi _1 = 1/\sigma ,$ which in turn implies that $y_1 = 0$.

Looking now for $x_2,$ we equate the ${\mathcal {O}}(\epsilon ^3)$ coefficients to zero:

B.30 $$\begin{aligned} 0&= x_0^2+x_0y_0+x_1y_{-1}+y_0^2+2y_{-1}y_1 \nonumber \\&\quad -\lambda y_1 -\mu (x_0y_1+x_1y_0+x_2y_{-1}+2y_{-1}y_2+2y_0y_1)\nonumber \\&\quad +(x_0y_2+x_1y_1+x_2y_0+x_3y_{-1}). \end{aligned}$$

which is also trivially satisfied as all terms either cancel with another or contain a factor of $x_0,y_{-1},$ or $y_0.$ Thus, we turn to ${\mathcal {O}}(\epsilon ^4)$ to determine $x_2.$ Equating the coefficients to zero gives

B.31 $$\begin{aligned} 0&= 2(x_0x_1+x_0y_1+x_1y_0+x_2y_{-1}+y_{-1}y_2+y_0y_1)-\lambda y_2 \nonumber \\&\quad -\mu \left( x_0y_2+x_1y_1+x_2y_0+x_3y_{-1}+y_1^2+2y_{-1}y_3+2y_0y_2\right) \nonumber \\&\quad +\lambda \sigma (x_0y_3+x_1y_2+x_2y_1+x_3y_0+x_4y_{-1}) \nonumber \\&= y_1(-\mu (x_1+y_1)+\lambda \sigma x_2). \end{aligned}$$

The solution we are interested in for $x_2$ comes from $y_1=0,$ which can be expressed in terms of $x_2$ as

B.32 $$\begin{aligned} 0 = \psi _{-1}(x_2-\phi _2), \end{aligned}$$

and thus

B.33 $$\begin{aligned} x_2 =\phi _2 = \frac{\delta _c+\mu -\sigma }{\lambda \sigma ^2}. \end{aligned}$$

At this point, we have a second-order expansion of the approximate equilibrium $x^*:$

B.34 $$\begin{aligned} x^* \approx \frac{1}{\sigma }\epsilon + \frac{\delta _c+\mu -\sigma }{\lambda \sigma ^2}\epsilon ^2+{\mathcal {O}}\left( \epsilon ^3\right) . \end{aligned}$$

Now, with the relation $w^* = \frac{\sigma }{\epsilon }x^*,$ we conclude that

B.35 $$\begin{aligned} w^* \approx 1+\frac{\delta _c+\mu -\sigma }{\lambda \sigma }\epsilon +{\mathcal {O}}\left( \epsilon ^2\right) . \end{aligned}$$
